# The efficacy of acupuncture for tension-type headache: a systematic review and meta-analysis of randomized controlled trials

**DOI:** 10.22514/jofph.2025.067

**Published:** 2025-12-12

**Authors:** Pen-Ting Lin, Sheng-Yu Su, Chia-Lung Shih

**Affiliations:** ^1^Department of Chinese Medicine, Ditmanson Medical Foundation Chia-Yi Christian Hospital, 600566 Chiayi, Taiwan; ^2^Clinical Research Center, Ditmanson Medical Foundation Chia-Yi Christian Hospital, 600566 Chiayi, Taiwan

**Keywords:** Acupuncture, Tension-type headache, Pain level, Meta-analysis

## Abstract

**Background**: Tension-type headache (TTH) is a common primary headache disorder characterized by bilateral, non-pulsating, mild-to-moderate pressing or tightening pain. The objective of this study was to investigate the efficacy of acupuncture for TTH using meta-analysis. **Methods**: We adhered to the PRISMA (Preferred Reporting Items for Systematic Reviews and Meta-Analyses) guidelines to conduct this research. A systematic search of multiple electronic databases, including PubMed, EMBASE, and the Cochrane Library, was conducted, covering all literature from their inception to August 2024. The articles investigating the efficacy of acupuncture for patients with TTH were included. Meta-analyses were used to pooled the effect size using RevMan 5.4. **Results**: A total of six randomized controlled trials (including 927 patients) were included. The results revealed the acupuncture group showing significant decrease in headache frequency at 6 weeks post-treatment (standardized mean difference (SMD) = −0.23; 95% confidence interval (CI): −0.43 to −0.03; *p* = 0.03), while the acupuncture group had higher odds of headache relief when compared with the sham-acupuncture group (odds ratio (OR) = 1.85; 95% CI: 1.34 to 2.57; *p* < 0.001). In subgroup analysis, acupuncture showed significant decrease in pain level compared to sham-acupuncture when patients received more than one month or 10 treatment sessions (SMD = −0.32; 95% CI: −0.56 to −0.09; *p* = 0.006). **Conclusions**: Our results suggest that acupuncture could be effective for TTH when the treatment period extends beyond one month or includes more than 10 sessions. **The PROSPERO Registration**: Registration number CRD42024602270.

## 1. Introduction

Tension-type headache (TTH) is the most common primary headache disorder and presents as bilateral, non-pulsating, mild-to-moderate pressing, or tightening pain [1]. It usually occurs without significant nausea or vomiting and is not aggravated by routine physical activity [1]. According to the 3rd edition of the International Classification of Headache Disorders (ICHD-3), TTH is divided into three subtypes: infrequent episodic, frequent episodic, and chronic TTH (Headache Classification Committee of the International Headache Society, 2018). Estimates indicate that TTH affects about 26.0% of people worldwide (95% CI: 22.7–29.5%) and occurs more frequently in women than in men [2]. A review of 568 heterogeneous studies found that TTH causes moderate disability, variable productivity losses, and severely reduced quality of life that significantly improved with treatment [3].

Currently, management of TTH combines both pharmacological and non-pharmacological strategies. Acute pharmacotherapy typically relies on simple analgesics—such as ibuprofen or acetaminophen—while preventive pharmacological options include low-dose tricyclic antidepressants (*e.g.*, amitriptyline) and other agents tailored to the patient’s comorbidities and headache frequency [4]. Non-drug interventions encompass physical modalities (*e.g.*, manual therapy, posture correction), psychological and behavioral therapies (including cognitive-behavioral therapy and biofeedback), relaxation techniques (such as progressive muscle relaxation and mindfulness) and complementary approaches like acupuncture or massage therapy [5, 6, 7].

Acupuncture, a cornerstone of Traditional Chinese Medicine, is increasingly employed in the management of TTH. Grounded in meridian theory and the regulation of Qi and blood, acupuncture involves the insertion of fine needles at specific acupoints to restore energy flow, harmonize physiological functions and relieve pain [8]. Common modalities include body acupuncture, auricular acupuncture, and scalp acupuncture—each of which has demonstrated distinct clinical benefits in TTH patients [5, 9, 10]. A growing body of randomized trials and systematic reviews confirms that acupuncture not only produces significant analgesia but also enhances overall quality of life in those suffering from TTH [5, 9, 10].

Meta-analyses indicate that acupuncture yields significant reductions in headache intensity and frequency, as well as improvements in quality of life for TTH sufferers [11, 12], although most trials to date have evaluated only short-term outcomes, leaving the durability of these benefits—and their trajectory at various post-treatment time points—largely uncharacterized. Furthermore, patient- and treatment-related factors that may modulate acupuncture’s efficacy remain poorly understood.

This meta-analysis aimed to quantify the therapeutic efficacy of acupuncture in TTH and to explore patient- and treatment-related factors that might modulate its clinical effectiveness.

## 2. Materials and methods

### 2.1 Search strategy

For this study, we adhered to the PRISMA (Preferred Reporting Items for Systematic Reviews and Meta-Analyses) guidelines (**Supplementary material 1**). The protocol was registered with the International Prospective Register of Systematic Reviews (PROSPERO) under registration number CRD42024602270. We conducted a systematic search of PubMed, EMBASE, and Cochrane Library from inception to August 2024; detailed search strategies are presented in **Supplementary Table 1**. To maximize comprehensiveness, we also manually reviewed the reference lists of retrieved articles as well as relevant grey literature, including conference proceedings, dissertations, and clinical trial records. Two authors (SYS and PTL) independently performed study selection where initially, duplicate articles among the search results were removed using EndNote, then the remaining articles were screened by reviewing their titles and abstracts for eligibility, and full texts of potentially eligible studies were assessed against our inclusion criteria. Discrepancies between the two reviewers were resolved through discussion until consensus was achieved.

### 2.2 Inclusion and exclusion criteria

We included articles that met the following criteria: (1) adult patients diagnosed with TTH according to ICHD-3 criteria; (2) intervention arm treated with manual acupuncture; (3) control arm treated with sham acupuncture; (4) randomized controlled trial (RCT) design; and (5) reporting of at least one of these outcomes: headache frequency, headache scores, analgesic usage or the proportion of patients achieving ≥50% reduction in headache severity. The exclusion criteria were as follows: (1) non-original articles (*e.g.*, reviews, editorials, protocols); (2) use of adjunct therapies (electroacupuncture, moxibustion, or other non-manual acupuncture modalities); (3) insufficient data reported for inclusion in meta-analysis; (4) patient populations with headache types other than TTH; and (5) lack of relevant outcomes (*e.g.*, pain intensity, attack frequency).

### 2.3 Outcome

Several outcomes were assessed to evaluate TTH relief. The primary outcome was headache frequency, defined as the number of days with headaches per month, while secondary outcomes included headache intensity, analgesic usage, and headache relief >50%. Headache score was measured using the visual analogue scale (VAS) or the numerical rating scale (NRS). This was measured at consultation and used the average score during the observation period. Analgesic usage, quantified as the total number of pain-relief medications taken per month, served as an additional indicator of whether acupuncture could reduce medication dependence. The proportion of patients achieving ≥50% headache relief was defined as the percentage of participants who experienced at least a 50% reduction in monthly headache days compared with baseline.

### 2.4 Data extraction

We used our developed format to extract data. The data included first author, publication year, sex ratio, sample size, mean age, treatment, outcomes, and follow-up periods. Two authors (SYS and PTL) independently extracted the data. Any disagreements were discussed by the two authors until consensus was reached.

### 2.5 Risk of bias assessment

The Cochrane Risk of Bias Tool version 2 (RoB 2.0) was used to evaluate the quality for each article. This assessment included five domains, including randomization processes, deviations from intended interventions, missing outcome data, measurement of the outcome, and selection of the reported result. Each domain was assessed to low risk, some concerns, and high risk, while overall risk was determined from the results of the five domains. A rating of “low risk” was given when all five domains were assessed as low risk; “Some concerns” was assigned when one domain showed some concerns while other domains were low risk; while “High risk” was assigned when any domain showed high risk or when two or more domains showed some concerns. The risk of bias was independently evaluated by two authors (SYS and PTL), with disagreements resolved through discussion.

### 2.6 Statistical analysis

All statistical analyses were conducted using RevMan 5.4 (Version 5.4, Nordic Cochrane Centre, The Cochrane Collaboration, Copenhagen, Denmark). For continuous data, standardized mean differences (SMDs) with 95% confidence intervals (CIs) were calculated for meta-analysis. For dichotomous data, odds ratio (OR) with 95% CIs was used. Heterogeneity was assessed using the *I^2^* statistic, where *I^2^* > 50% indicated significant heterogeneity. A random-effects model was used to pool the effect size regardless of heterogeneity. Subgroup analyses were conducted to explore differences in acupuncture efficacy at various time points or other factors. A sensitivity analysis was performed to determine whether individual RCTs influenced the pooled estimates.

## 3. Results

### 3.1 Search strategy and major characteristics of the included articles

The detailed article screening procedure is shown in Fig. 1. We identified 982 articles from the three databases, and after removing duplicates, 524 articles remained. Screening of titles and abstracts yielded 34 relevant articles, and following full-text review, 6 articles met our inclusion criteria [13, 14, 15, 16, 17, 18]. The major characteristics of the 6 articles (including 927 patients) are shown in Table 1 (Ref. [13, 14, 15, 16, 17, 18]). Between 2000 and 2022, six RCTs enrolled adults (mean age 36.0–50.4 years; 50–82% female) suffering from chronic TTH, with two studies also including episodic TTH. Sample sizes ranged from 15 to 209 participants for each group. Acupuncture interventions comprised 6–20 sessions of manual needling (20–30 minutes each), while sham-acupuncture controls used either superficial needling at non-acupoints (avoiding Deqi or active trigger points) or non-penetrating placebo needles at the same locations. Key outcomes across studies included headache frequency and duration, pain intensity, response rates, and use of acute medication or daily analgesics.

**Fig. 1.** Flowchart of the article screening process.

**Table 1.** Major characteristics of included articles.

Study	Treatment	Type of headache	Age (yr) (mean ± SD)	Female (%)	Sample size	Sessions, n Time per session (min)	Treatment type of SA	Key outcome measurements
Zheng 2022 [15]	Acupuncture	CTTH	43.0 ± 12.5	75	110	20 sessions and 30 min each session	Superficial needling at the same acupoints to avoid achieving “Deqi”	Response rate, number of headache days, headache intensity, use of acute medication
	Sham-acupuncture		43.2 ± 12.8	69	108			
Gildir 2019 [13]	Acupuncture	CTTH	36.7 ± 7.6	41	80	6 sessions and 20 min each session	There are no areas of adipose tissue where active TrP is present.	Headache intensity, frequency and duration of headache, short form-36
	Sham-acupuncture		36.0 ± 8.3	44	80			
Kwak 2008 [17]	Acupuncture	CTTH	45.05 ± 12.57	82	17	8 sessions and 25 min each session	non-acupoints in the same regions	VAS, HDI, six-point Likert Scale, algometer score (Rt, Lt)
	Sham-acupuncture		49.4 ± 11.14	80	15			
Endres 2007 [16]	Acupuncture	CTTH & ETTH	39.2 ± 11.4	78	209	10 sessions and 30 min each session	Superficially needling at non-acupoints to avoid achieving “Deqi”	Response rate, number of headache days per four weeks, Von Korff chronic pain grade scale, SF-12, medication use
	Sham-acupuncture		38.9 ± 12.2	79	200			
Karst 2001 [14]	Acupuncture	CTTH & ETTH	47.9 ± 13.8	50	34	10 sessions and 30 min each session	Non-penetrating placebo needle at the same acupoints	Headache frequency and intensity (VAS), site, and duration of headache attacks.
	Sham-acupuncture		48.2 ± 14.6	60	35			
Karst 2000 [18]	Acupuncture	CTTH	50.4 ± 13.5	38	21	10 sessions and 30 min each session	Non-penetrating placebo needle at the same acupoints	Pressure pain thresholds, daily consumption of analgesics, pain intensity, duration, and frequency of headache attacks
	Sham-acupuncture		47.3 ± 16.5	61	18			

*SD: standard deviation; min: minute; SA: sham acupuncture; CTTH: chronic tension-type headache; ETTH: episodic tension-type headache; TrP: trigger points; VAS: visual analog scale; HDI: headache disability inventory; SF-12: short form 12 health survey.*

### 3.2 Risk of bias assessment

The assessment of risk of bias is shown in Fig. 2. Of the six articles, three were assessed as high risk and three as low risk. The major reason for high risk of bias was due to most domains having some concerns.

**Fig. 2.** Risk of bias assessment for individual articles (A) and overall risk of bias across domains (B).

### 3.3 Meta-analysis

#### 3.3.1 Headache frequency

Four articles reported headache frequency. There was no significant difference in headache frequency between acupuncture and sham acupuncture groups within 12 weeks post-treatment (SMD = −1.12; 95% CI: −2.76 to 0.52; *p* = 0.18) (Fig. 3A). No significant difference between groups was observed at the end of treatment (SMD = −0.81; 95% CI: −1.74 to 0.12; *p* = 0.09) (Fig. 3B); however, the acupuncture group showed a significant decrease in headache frequency at 6 weeks post-treatment (SMD = −0.23; 95% CI: −0.43 to −0.03; *p* = 0.03) (Fig. 3C). We further divided the sham acupuncture group into two subgroups of acupoint or active trigger-point needling and non-acupoint or inactive trigger-point needling, but no significant difference in headache frequency between these two groups was observed within 12 weeks after treatment (Fig. 3A).

**Fig. 3.** Comparison of headache frequency between the acupuncture and sham-acupuncture groups at subgroup analyses (Acupoint *vs*. Non-Acupoint/Non-Trigger Point Needling) within 12 weeks post-treatment (A), the end of treatment (B), and 6 weeks post-treatment (C). SD: standard deviation; Std: standardized; CI: confidence interval; IV: inverse variance.

#### 3.3.2 Pain level

Two articles reported pain level after treatment, although there was no significant difference in pain level between the acupuncture and sham-acupuncture groups after immediate treatment (SMD = −0.06; 95% CI: −0.43 to 0.32; *p* = 0.77) (Fig. 4A), nor following six weeks post-treatment (SMD = 0.06; 95% CI: −0.31 to 0.44; *p* = 0.74) (Fig. 4B).

**Fig. 4.** Comparison of headache score (VAS at consultation) between acupuncture and sham-acupuncture groups at immediate post-treatment (A), and 6 weeks after treatment (B). SD: standard deviation; Std: standardized; CI: confidence interval; IV: inverse variance.

We investigated the difference in pain level between two groups at different follow-up periods; however, no significant difference in pain levels between the two groups was observed at the end of treatment or at the 4-week, 8-week, and 12-week follow-up periods (Fig. 5).

**Fig. 5.** Comparison of headache score (mean VAS) between acupuncture and sham-acupuncture groups at the end of treatment (A), 4 weeks (B), 8 weeks (C), and 12 weeks (D) follow-up visits. SD: standard deviation; Std: standardized; CI: confidence interval; IV: inverse variance.

#### 3.3.3 Analgesic usage

Two articles reported analgesic usage after treatment. There was no significant difference in analgesic usage between the acupuncture and sham-acupuncture groups (OR = −0.06; 95% CI: −0.51 to 0.39; *p* = 0.80) at the end of treatment and at six weeks post-treatment (OR = −0.36; 95% CI: −0.74 to 0.02; *p* = 0.06) (Fig. 6).

**Fig. 6.** Comparison of analgesic usage between acupuncture and sham-acupuncture groups at the end of treatment (A) and 6 weeks (B) follow-up visits. SD: standard deviation; Std: standardized; CI: confidence interval; IV: inverse variance.

#### 3.3.4 Headache relief >50%

Two articles reported headache relief >50%. The acupuncture group had higher odds of headache relief when compared with the sham-acupuncture group (OR = 1.85; 95% CI: 1.34 to 2.57; *p* = 0.0002) (**Supplementary Fig. 1**).

#### 3.3.5 Subgroup analysis

The articles that reported pain level were subgrouped for meta-analysis. We divided the sham-acupuncture group into acupoint or active trigger-point needling and non-acupoint or inactive trigger-point needling groups respectively, and found that acupuncture revealed significant decrease in pain level when compared with acupoint or active trigger-point needling (SMD = −0.32; 95% CI: −0.56 to −0.09; *p* = 0.006) (**Supplementary Fig. 2A**); however, no significant difference in pain level between the acupuncture and non-acupoint or inactive trigger-point needling groups was observed (SMD = −2.42; 95% CI: −7.32 to 2.52; *p* = 0.34) (**Supplementary Fig. 2A**).

We divided the analysis into two subgroups based on the treatment duration or treatment sections (**Supplementary Fig. 2B,C**). Acupuncture showed a significant decrease in pain level compared to sham-acupuncture when patients received more than 10 treatment sections (SMD = −0.32; 95% CI: −0.56 to −0.09; *p* = 0.006) or one month (SMD = −0.32; 95% CI: −0.56 to −0.09; *p* = 0.006) (**Supplementary Fig. 2B,C**); however, no significant difference emerged in treatments with fewer than one month or less than 10 treatment sections (**Supplementary Fig. 2B,C**). We further divided the patients into two groups: those from East Asian countries and those from non-East Asian countries (**Supplementary Fig. 2D**), but found no significant difference in pain levels between acupuncture and sham-acupuncture groups for either geographical group. We also divided the patients into two groups: those with chronic TTH and those with chronic or episodic TTH (**Supplementary Table 2**), but found no significant difference in headache frequency between acupuncture and sham-acupuncture groups was observed in both types of TTH, while TTH type did not affect the headache frequency after receiving acupuncture. Patients were also stratified by age into those younger than 45 years and those 45 years or older (**Supplementary Table 2**), but no significant difference in headache frequency between acupuncture and sham-acupuncture groups for either age group was found. Headache frequency following acupuncture did not differ between patients younger than 45 years and those 45 years or older (**Supplementary Table 2**).

#### 3.3.6 Sensitivity analysis

We performed sensitivity analysis by removing one article at a time. In removing the article reported by Gildir *et al*. [13] 2019, we found that the acupuncture group had significant decrease in headache frequency when compared with the sham-acupuncture group at the end of treatment (**Supplementary Table 3**); while upon removing the article reported by Karst *et al*. [14] 2001, the acupuncture group revealed significant decrease in headache frequency when compared with the sham-acupuncture group at 6 week post-treatment (**Supplementary Table 3**), although for pain level, we did not observe any significant difference in pain level between the two groups in the sensitivity analysis (**Supplementary Table 4**). After excluding studies with a high risk of bias, the pooled effect size remained unchanged (**Supplementary Tables 3 and 4**).

## 4. Discussion

In this study, we performed a meta-analysis to investigate the efficacy of acupuncture for TTH. Our meta-analysis found no overall significant difference in headache frequency between acupuncture and sham groups, although at the 6-week follow-up, acupuncture revealed significant decrease in headache frequency compared with sham-acupuncture. In terms of pain intensity, acupuncture produced a significantly greater decrease than acupoint or active trigger-point needling, but for analgesic usage, no significant differences were observed between acupuncture and sham-acupuncture groups. Acupuncture had higher odds in pain relief greater than 50% when compared with sham-acupuncture; moreover, acupuncture resulted in significantly lower pain levels when treatment lasted longer than one month or included more than 10 sessions. In sensitivity analysis, after excluding specific articles, acupuncture showed a significant decrease in pain levels compared with sham-acupuncture.

Acupuncture has been found to be effective in managing pain conditions such as migraines [19] and knee osteoarthritis [20]. The mechanisms of acupuncture encompass both peripheral and central nervous system pathways. Peripherally, it influences the release of adenosine triphosphate, modulates Transient Receptor Potential Vanilloid channel activity, and regulates immune responses [21, 22]. Centrally, acupuncture alters the levels of key neurotransmitters—including opioids, serotonin, and glutamate—which play vital roles in modulating pain perception and various physiological functions [21, 22]. A recent meta-analysis examined the effectiveness of acupuncture for TTH [12]. In comparison with sham-acupuncture, only two post-treatment outcomes were considered: TTH frequency and responder rate [12]. They concluded that acupuncture significantly decreased TTH frequency but showed no significant difference in responder rate [12]. Another meta-analysis reported the effect of acupuncture on TTH [9]. While it included 30 RCTs, the analysis combined studies involving both electroacupuncture and manual acupuncture [9]. In the subgroup analysis, the authors reported that electroacupuncture produced a greater reduction in headache frequency compared to manual acupuncture, suggesting a potentially enhanced therapeutic effect with electrical stimulation [9]. However, our study focused on the effect of manual acupuncture. Another previous study also demonstrated the sustained effect of acupuncture for TTH [23]. However, only five RCTs directly compared acupuncture with sham acupuncture [23]. Although the study assessed acupuncture’s effectiveness across various follow-up periods—from immediately after treatment completion to six months post-treatment—most time points were represented by only a single RCT, limiting the strength and generalizability of the conclusions. However, they did not perform additional subgroup analyses to explore whether other factors might influence the efficacy of acupuncture.

In this study, we further investigated the impact of follow-up period on TTH frequency and found that acupuncture demonstrated significant improvement in TTH frequency only at 6 weeks post-treatment (Fig. 3). In addition, we classified sham-acupuncture into two groups: acupoint/acute trigger-point needling and non-acupoint/non-acute trigger-point needling. The results showed that the type of sham-acupuncture did not affect TTH frequency when compared with acupuncture (Fig. 3A). However, our results demonstrated that acupuncture had significant increase in odds of patients with headache relief >50% (**Supplementary Fig. 1**). Our results provide a more accurate assessment of acupuncture’s efficacy for TTH.

In this study, we found that acupuncture significantly reduced headache frequency at the six-week follow-up compared to sham acupuncture (Fig. 3C). Although statistically significant, the SMD of 0.23 indicates a modest clinical effect. This degree of improvement may still be clinically relevant in certain contexts—particularly when alternative treatments are limited or when acupuncture is used as part of a multimodal therapeutic approach. However, for broader clinical implementation, this finding should be interpreted with caution and ideally supported by additional patient-centered outcomes such as quality of life or satisfaction. Furthermore, we observed that acupuncture was associated with an OR of 1.85 for achieving greater than 50% headache relief compared to sham acupuncture (**Supplementary Fig. 1**). Given that an OR greater than 1.20 is generally considered clinically meaningful, this result suggests that acupuncture provides a substantial benefit in terms of symptom reduction. Collectively, these findings support the clinical relevance of acupuncture in the management of headache disorders.

The duration of acupuncture treatment plays a crucial role in determining its effectiveness for TTH. This relationship between treatment duration and therapeutic outcomes has been well-documented in related conditions. Specifically, according to a comprehensive systematic review, patients experiencing migraines typically require an extended treatment period of at least 10 weeks of acupuncture therapy to achieve optimal therapeutic benefits [24]. This finding aligns with our own research observations, where we discovered a statistically significant reduction in pain levels among participants who underwent either extended treatment periods exceeding one month or completed more comprehensive treatment courses consisting of more than 10 individual acupuncture sessions (**Supplementary Fig. 2B**). These results strongly suggest that the therapeutic benefits of acupuncture for TTH are time-dependent and may require sustained treatment engagement to maximize efficacy.

Across the six RCTs, acupuncture differed not only in session number and duration but in how needles were manipulated as well, including where they were placed, and whether the characteristic Deqi sensation was elicited (Table 1). Superficial or non-penetrating needling at non-acupoints—used in most sham controls—minimizes tissue stimulation and Deqi, likely curbing the release of endogenous opioids and thus producing only marginal, placebo-mediated benefit [25, 26]. In contrast, trigger-point needling directly targets hyperirritable myofascial nodules and has been shown to raise pressure-pain thresholds and reduce headache intensity more robustly [13, 27]. The attainment of Deqi—a composite sensation of numbness, heaviness, or radiating warmth—has been linked to better clinical outcomes in both systematic reviews and experimental studies, underscoring the need for its consistent reporting [28, 29]. Finally, electroacupuncture, by providing continuous electrical stimulation, appears to augment manual needling through sustained afferent input and increased endogenous analgesic signaling, which might explain its superiority in some pain conditions [26]. Future trials should standardize and fully describe these technical parameters to enhance reproducibility and optimize therapeutic effect.

### 4.1 Limitations

There are some limitations in this study. Firstly, although we attempted to classify outcomes into different follow-up periods to investigate factors affecting the efficacy of acupuncture for TTH, the number of articles included for meta-analysis was rather small, indicating the possibility of unreliable results; and secondly, we found that treatment duration and number of treatment sessions affected the efficacy of acupuncture. Furthermore, we were unable to determine the optimal treatment period or number of sessions that would maximize efficacy. Future studies should consider longer follow-up periods to evaluate the sustained effects of acupuncture on TTH and explore its potential long-term mechanisms of action. Finally, despite our comprehensive search strategy, some eligible studies may not have been included in our analysis due to database search limitations.

### 4.2 Implications

Our findings highlight the importance of treatment duration and session frequency in maximizing the efficacy of acupuncture for TTH. Given that significant pain reduction was observed only when treatment extended beyond one month or included more than 10 sessions, clinicians should consider longer treatment courses for optimal patient outcomes. Future clinical guidelines should recommend standardized treatment protocols that account for both session frequency and follow-up duration to enhance therapeutic benefits. Additionally, further RCTs are needed to establish the ideal treatment duration and number of sessions required to achieve sustained pain relief in TTH patients.

## 5. Conclusions

While this comprehensive meta-analysis suggests that acupuncture could reduce headache frequency and pain intensity in patients with TTH, these findings must be interpreted with caution given the small number of included trials, modest sample size, and the risk of bias concerns in several studies. We observed a statistically significant reduction in headache frequency at the 6-week follow-up and greater odds of achieving >50% pain relief with acupuncture versus sham treatment. A secondary analysis also indicated more pronounced pain reduction when treatment extended beyond one month or exceeded ten sessions; nevertheless, these results are preliminary, and further well-designed, large-scale randomized controlled trials are required to validate the efficacy and long-term benefits of acupuncture for TTH.

## Supplementary Material

Supplementary material associated with this article can be found, in the online version, at https://files.jofph.com/files/article/1999363108500324352/attachment/Supplementary%20material.zip.




