# Supplementary material for: The efficacy of acupuncture for tension-type headache: a systematic review and meta-analysis of randomized controlled trials
Source: J Oral Facial Pain Headache. 2025 Dec 12;39(4):60–9. doi: 10.22514/jofph.2025.067 (PMC12727187; doi:10.22514/jofph.2025.067)
Supplement: Supplementary file 1 [file Supplementary-material-1.docx]

Supplementary material 1

PRISMA-A checklist

| **Check** | **Subjects** | **PRISMA for Acupuncture** |
| --- | --- | --- |
| ***Title*** | | |
| **Page 1** | **Title** | 1* Identify the report as a systematic review, meta-analysis, or both; if applicable, state the specific type of acupuncture treatment, such as manual acupuncture or electroacupuncture. |
| ***Abstract*** | | |
| **Page 1** | **Structured summary** | 2^†^ Provide a structured summary including, as applicable: background; objectives; data sources; study eligibility criteria, participants, and interventions; study appraisal and synthesis methods; results limitations; conclusions and implications of key findings; systematic review registration number. |
| ***Introduction*** | | |
| **Page 2** | **Rationale** | 3* Describe the rationale for what is already known about acupuncture for the target condition in the background; if applicable, state what is already known about the specific types of acupuncture to be studied, and describe whether there is any difference of the effects among different types of acupuncture. |
| **Page 2** | **Objectives** | 4^†^ Provide an explicit statement of questions being addressed with reference to participants, interventions, comparisons, outcomes, and study design (PICOS) |
| ***Methods*** | | |
| **Page 2** | **Protocol and registration** | 5^†^ Indicate if a review protocol exists, if and where it can be accessed (*e.g.*, web address), and, if available, provide registration information including registration number. |
| **Page 3** | **Eligibility criteria** | 6^†^ Specify study characteristics (*e.g.*, PICOS, length of follow-up) and report characteristics (*e.g.*, years considered, language, publication status) used as criteria for eligibility, giving rationale.  6a.1^‡^ Describe the diagnostic criteria of the target condition in Western medicine.  6a.2^‡^ If applicable, describe the diagnostic criteria in terms of Traditional Medicine, such as Traditional Chinese Medicine.  6b^‡^ Describe the types of acupuncture to be included, such as traditional acupuncture, electroacupuncture, or fire acupuncture.  6c^‡^ If applicable, report measures for therapeutic effects using the terminology of either traditional medicine (*e.g.*, syndrome score for syndrome remission) or Western medicine (*e.g.*, pain intensity). |
| **Page 5** | **Information sources** | 7* Describe all sources of information (*e.g.*, databases with dates of coverage, contact with study authors to identify additional studies) in the search, and report the date of the last search. If applicable, report the databases or complementary search methods for acupuncture or traditional medicine. |
| **Supplementary Table 1** | **Search** | 8* Present full electronic search strategy for at least one commonly used database (*e.g.*, MEDLINE), including any limits used, such that it could be repeated. If applicable, include the full search strategy for at least a Western and a traditional medicine database for each systematic review where both were used. |
| **Page 5** | **Study selection** | 9^†^ State the process for selecting studies (*i.e.*, screening, eligibility, included in systematic review, and, if applicable, included in the meta-analysis). |
| **Page 6–7** | **Data collection**  **process** | 10^†^ Describe method of data extraction from reports (*e.g.*, piloted forms, independently, in duplicate) and any processes for obtaining and confirming data from investigators. |
| **Page 7** | **Data items** | 11* List and define all variables for which data were sought (*e.g.*, PICOS, funding sources) and any assumptions and simplifications made; describe data items about details of acupuncture interventions and controls (*e.g.*, sham acupuncture) referring to TIDieR when applicable. |
| **Page 7** | **Risk of bias in**  **Individual studies** | 12^†^ Describe methods used for assessing risk of bias of individual studies (including specification of whether this was done at the study or outcome level), and how this information is to be used in any data synthesis. |
| **Page 7–8** | **Summary measures** | 13^†^ State the principal summary measures (*e.g.*, risk ratio, difference in means). |
| **Page 7–8** | **Synthesis of results** | 14^†^ Describe the methods of handling data and combining results of studies, if done, including measures of consistency (*e.g.*, *I*^2^) for each meta-analysis. |
| **Page 7** | **Risk of bias across studies** | 15^†^ Specify any assessment of risk of bias that may affect the cumulative evidence (*e.g.*, publication bias, selective reporting within studies). |
| **Page 8** | **Additional analyses** | 16^†^ Describe methods of additional analyses (*e.g.*, sensitivity or subgroup analyses, meta-regression), if done, indicating which were pre-specified. |
| ***Results*** | | |
| **Fig. 1** | **Study selection** | 17^†^ Give numbers of studies screened, assessed for eligibility, and included in the review, with reasons for exclusions at each stage, ideally with a flow diagram. |
| **Page 8** | **Study characteristics** | 18* For each study, present characteristics that were extracted (*e.g.*, study size, PICOS, follow-up period) and provide the citations of the included studies. Summarize details of the acupuncture intervention for each study in a table referring to TIDieR.  18a^‡^ Describe details of “De-qi” after acupuncture reported in the included studies. |
| **Page 9 and Fig. 2** | **Risk of bias within**  **studies** | 19^†^ Present data on risk of bias of each study and, if available, any outcome-level assessment (see item 12). |
| **Page 9–10** | **Results of individual**  **studies** | 20^†^ For all outcomes considered (benefits or harms), present, for each study: (a) simple summary data for each intervention group and (b) effect estimates and confidence intervals, ideally with a forest plot. |
| **Page 9–10** | **Synthesis of results** | 21^†^ Present results of each meta-analysis done, including confidence intervals and measures of consistency. |
| **Page 12 & Supplementary Tables 3 and 4** | **Risk of bias across**  **studies** | 22^†^ Present results of any assessment of risk of bias across studies (see item 15). |
| **Page 12** | **Additional analysis** | 23^†^ Give results of additional analyses, if done (*e.g.*, sensitivity or subgroup analyses, meta-regression (see item 16)). |
| ***Discussion*** | | |
| **Page 13** | **Summary of evidence** | 24^†^ Summarize the main findings including the strength of evidence for each main outcome; consider their relevance to key groups (*e.g.*, health care providers, users, and policy  makers). |
| **Page 16** | **Limitations** | 25^†^ Discuss limitations at study and outcome level (*e.g.*, risk of bias), and at review level (*e.g.*, incomplete retrieval of identified research, reporting bias). |
| **Page 17** | **Conclusions** | 26^†^ Provide a general interpretation of the results in the context of other evidence, and implications for future research. |
| ***Funding*** | | |
| **Page 18** | **Funding** | 27^†^ Describe sources of funding for the systematic review and other support (*e.g.*, supply of data); role of funders for the systematic review. |

Note: *modified original item. ^†^unmodified item from PRISMA. ^‡^new extended item.

PICOS, participants, interventions, comparisons, outcomes, and study design.
