# Supplementary material for: The efficacy of acupuncture for tension-type headache: a systematic review and meta-analysis of randomized controlled trials
Source: J Oral Facial Pain Headache. 2025 Dec 12;39(4):60–9. doi: 10.22514/jofph.2025.067 (PMC12727187; doi:10.22514/jofph.2025.067)
Supplement: Supplementary file 2 [file Supplementary-material-2.docx]

Supplementary material 2


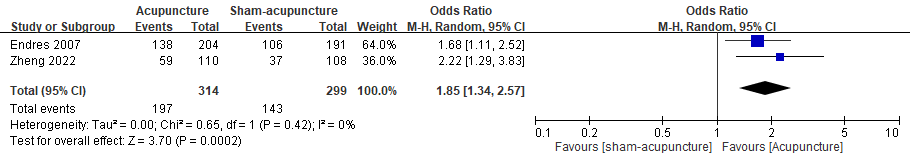


**Supplementary Fig. 1. Number of participants achieving >50% headache relief at treatment completion in the acupuncture versus sham-acupuncture groups.** CI: confidence interval; IV: inverse variance; M-H: Mantel–Haenszel method.


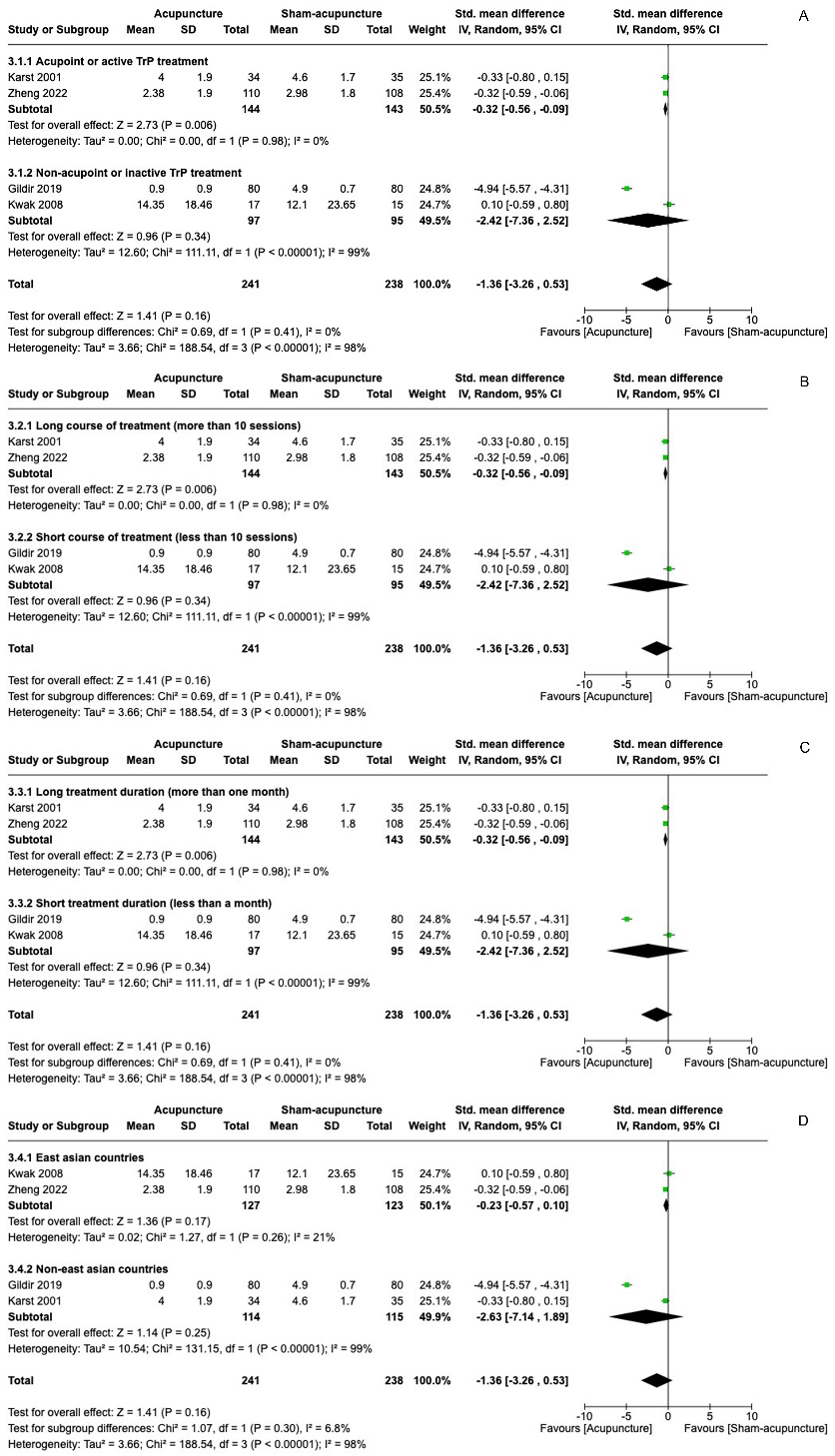


**Supplementary Fig. 2. Subgroup analyses of headache scores between the acupuncture and sham-acupuncture groups within 12 weeks post-treatment: (A) acupoint *vs*. non-acupoint/non-trigger-point needling; (B) number of treatment sessions; (C) treatment duration; and (D) regional differences (East Asia *vs*. non-East Asia).** SD: standard deviation; min: minute; CI: confidence interval; IV: inverse variance.

Supplementary Table 1. The detailed search strategies for each database.

| Search number | | Query |
| --- | --- | --- |
| PubMed | | |
|  | #9 | #2 AND #8 |
|  | #8 | #7 OR #5 |
|  | #7 | #1 AND #6 |
|  | #6 | #3 OR #4 |
|  | #5 | tension-type headache [MeSH Terms] |
|  | #4 | “tension” [Title/Abstract] |
|  | #3 | “tension-type” [Title/Abstract] |
|  | #2 | “acupuncture” [Title/Abstract] OR “electroacupuncture” [Title/Abstract] OR “electro-acupuncture” [Title/Abstract] OR “acupuncture” [MeSH Terms] OR “acupuncture therapy” [MeSH Terms] |
|  | #1 | “headache” [Title/Abstract] OR “head pain” [Title/Abstract] OR “head ache” [Title/Abstract] OR “cephalalgia” [Title/Abstract] OR “cephalgia” [Title/Abstract] OR “migraine” [Title/Abstract] OR “cephalodynia” [Title/Abstract] OR “cranial pain” [Title/Abstract] OR “hemicranias” [Title/Abstract] OR “cerebral pain” [Title/Abstract] OR “cranialgca” [Title/Abstract] OR “headache” [MeSH Terms] |
| Embase | | |
|  | #14 | #7 AND #13 |
|  | #13 | #12 OR #10 |
|  | #12 | #3 AND #11 |
|  | #11 | #8 OR #9 |
|  | #10 | tension-type headache'/exp |
|  | #9 | tension': ab, ti |
|  | #8 | tension-type': ab, ti |
|  | #7 | #4 OR #5 OR #6 |
|  | #6 | acupuncture'/exp |
|  | #5 | acupuncture therapy'/exp |
|  | #4 | acupuncture': ab, ti OR “electroacupuncture”: ab, ti OR “electro-acupuncture”: ab, ti |
|  | #3 | #1 OR #2 |
|  | #2 | headache'/exp |
|  | #1 | headache': ab, ti OR “head pain”: ab, ti OR “head ache”: ab, ti OR “cephalalgia”: ab, ti OR “cephalgia”: ab, ti OR “migraine”: ab, ti OR “cephalodynia”: ab, ti OR “cranial pain”: ab, ti OR “hemicranias”: ab, ti OR “cerebral pain”: ab, ti OR “cranialgia”: ab, ti |
| Cochrane Library | | |
|  | #1 | headache OR head pain OR head ache OR cephalalgia OR cephalgia OR migraine OR cephalodynia OR cranial pain OR hemicranias OR cerebral pain OR cranialgca |
|  | #2 | MeSH descriptor: [Headache] explode all trees |
|  | #3 | #1 OR #2 |
|  | #4 | acupuncture OR electroacupuncture OR electro-acupuncture |
|  | #5 | MeSH descriptor: [Acupuncture] explode all trees |
|  | #6 | MeSH descriptor: [Acupuncture Therapy] explode all trees |
|  | #7 | #4 OR #5 OR #6 |
|  | #8 | tension-type |
|  | #9 | tension |
|  | #10 | MeSH descriptor: [Tension-Type Headache] explode all trees |
|  | #11 | #8 OR #9 |
|  | #12 | #3 AND #11 |
|  | #13 | #12 OR #10 |
|  | #14 | #7 AND #13 |

MeSH: Medical Subject Headings.

Supplementary Table 2. Subgroup analyses conducted within the 12-week post-treatment period compared headache frequency in the acupuncture.

| Subgroup | No of articles | SMD | 95% CI | *p*-value |
| --- | --- | --- | --- | --- |
| Older than 45 years | 2 | 0.00 | −0.37 to 0.38 | 0.99 |
| Young than 45 years | 2 | −2.25 | −6.05 to 1.55 | 0.25 |
| Test for subgroup differences |  |  |  | 0.18 |
| CTTH and ETTH | 2 | −0.23 | −0.51 to 0.04 | 0.10 |
| CTTH | 2 | −2.10 | −6.22 to 2.03 | 0.32 |
| Test for subgroup differences |  |  |  | 0.18 |

SMD: standardized mean difference; CI: confidence interval; CTTH: chronic tension-type headache; ETTH: episodic tension-type headache.

Supplementary Table 3. Sensitivity analysis for headache frequency.

| Subgroup | Effect size | | | | Study removed |
| --- | --- | --- | --- | --- | --- |
|  | No. of articles | SMD | 95% CI | *p*-value |  |
| The end of treatment | | | | | |
|  | 3 | −0.97 | −2.24 to 0.29 | 0.13 | Karst 2001 |
|  | 3 | −0.92 | −2.09 to 0.25 | 0.12 | Karst 2000 |
|  | 3 | −0.32 | −0.50 to −0.15 | **0.0003** | Gildir 2019 |
|  | 3 | −0.98 | −2.27 to 0.31 | 0.14 | Endres 2007 |
|  | 2 | −1.22 | −3.02 to 0.57 | 0.18 | Articles with high risk of bias |
| 6 weeks post-treatment | | | | | |
|  | 2 | −0.29 | −0.48 to −0.10 | **0.003** | Karst 2001 |
|  | 2 | −0.23 | −0.51 to 0.04 | 0.10 | Karst 2000 |
|  | 2 | 0.00 | −0.37 to 0.38 | 0.99 | Endres 2007 |

SMD: standardized mean difference; CI: confidence interval. Bolded numbers indicate *p*-values < 0.05.

Supplementary Table 4. Sensitivity analysis for headache score.

| Subgroup | Effect size | | | | Study removed |
| --- | --- | --- | --- | --- | --- |
|  | No. of articles | SMD | 95% CI | *p*-value |  |
| The end of treatment | | | | | |
|  | 3 | −1.71 | −5.04 to 1.62 | 0.32 | Zheng 2022 |
|  | 3 | −1.56 | −4.32 to 1.20 | 0.27 | Kwak 2008 |
|  | 3 | −1.7 | −4.93 to 1.52 | 0.30 | Karst 2001 |
|  | 3 | 0.18 | −0.04 to 0.40 | 0.11 | Gildir 2019 |
|  | 2 | −2.46 | −7.74 to 2.81 | 0.36 | Articles with high risk of bias |
| 4 weeks after treatment | | | | | |
|  | 2 | −2.46 | −7.33 to 2.41 | 0.32 | Zheng 2022 |
|  | 2 | −2.42 | −7.34 to 2.49 | 0.33 | Kwak 2008 |
|  | 2 | 0.07 | −0.18 to 0.32 | 0.57 | Gildir 2019 |
|  | 2 | −2.42 | −7.34 to 2.49 | 0.33 | Articles with high risk of bias |

SMD: standardized mean difference; CI: confidence interval.
